# Essential role for autophagy protein VMP1 in maintaining neuronal homeostasis and preventing axonal degeneration

**DOI:** 10.1038/s41419-021-03412-5

**Published:** 2021-01-22

**Authors:** Panpan Wang, Xi Chen, Yuanyuan Wang, Congcong Jia, Xinyao Liu, Ying Wang, Haifeng Wu, Huaibin Cai, Han-Ming Shen, Weidong Le

**Affiliations:** 1 Liaoning Provincial Key Laboratory for Research on the Pathogenic Mechanisms of Neurological Diseases, the First Affiliated Hospital, Dalian Medical University, Dalian, 116011 China; 2 Transgenic Section, Laboratory of Neurogenetics, National Institute on Aging, National Institutes of Health, Bethesda, MD 20892 USA; 3 Faculty of Health Sciences, University of Macau, Macau, 999078 SAR China; 4 Institute of Neurology and Department of Neurology, Sichuan Academy of Medical Sciences-Sichuan Provincial Hospital, Medical School of UETSC, Chengdu, 610072 China

**Keywords:** Neuroscience, Pathogenesis

## Abstract

Vacuole membrane protein 1 (VMP1), the endoplasmic reticulum (ER)-localized autophagy protein, plays a key role during the autophagy process in mammalian cells. To study the impact of *VMP1*-deficiency on midbrain dopaminergic (mDAergic) neurons, we selectively deleted *VMP1* in the mDAergic neurons of *VMP1*^fl/fl^/*DAT*^CreERT2^ bigenic mice using a tamoxifen-inducible *CreERT2/loxp* gene targeting system. The *VMP1*^fl/fl^/*DAT*^CreERT2^ mice developed progressive motor deficits, concomitant with a profound loss of mDAergic neurons in the substantia nigra pars compacta (SNc) and a high presynaptic accumulation of α-synuclein (α-syn) in the enlarged terminals. Mechanistic studies showed that *VMP1* deficiency in the mDAergic neurons led to the increased number of microtubule-associated protein 1 light chain 3-labeled (LC3) puncta and the accumulation of sequestosome 1/p62 aggregates in the SNc neurons, suggesting the impairment of autophagic flux in these neurons. Furthermore, *VMP1* deficiency resulted in multiple cellular abnormalities, including large vacuolar-like structures (LVSs), damaged mitochondria, swollen ER, and the accumulation of ubiquitin^+^ aggregates. Together, our studies reveal a previously unknown role of VMP1 in modulating neuronal survival and maintaining axonal homeostasis, which suggests that *VMP1* deficiency might contribute to mDAergic neurodegeneration via the autophagy pathway.

## Introduction

Vacuole membrane protein 1 (VMP1), an endoplasmic reticulum (ER)-resident protein, has attracted attention recently owing to its essential role on mediating autophagy. *VMP1* gene encodes a 406-amino-acid transmembrane protein containing six hydrophobic regions^1^. Of them, an autophagy-related (ATG) domain is localized in the middle of the chain^2^. Once mutated at the ATG domain *VMP1* fails to induce microtubule-associated protein 1 light chain 3 (LC3) recruitment and loses the interaction with Beclin-1, eventually blocking autophagy initiation^2^. However, recent work demonstrates that the *VMP1* depletion reversely promotes the accumulation of LC3-labeled autophagic structures at ER in the *VMP1* knockout (KO) mammalian cells upon aberrant nutrient conditions^3^, suggesting that VMP1 may play an important role in the autophagic process by regulating interactions between ER and autophagic-isolation membrane^4^. These studies indicate that *VMP1* deficiency inhibits autophagosome maturation, disrupts the association with ER and blocks the fusion with lysosome^4^. Besides the animal studies, the research in *Dictyostelium*^5^, plants^6^, and *Chlamydomonas*^7^ also indicates that VMP1 is highly involved in the processes of protein secretion, phagocytosis, osmoregulation, and cytokinesis to mediate the diverse cellular process.

The autophagy-lysosome pathway is responsible for the clearance of intracellular protein aggregates, the dysfunction of which is associated with several neurodegenerative diseases, such as Parkinson’s disease (PD), Alzheimer’s disease, amyotrophic lateral sclerosis, and Huntington’s disease^8^. So far, several mice models, such as *Atg5*^fl/fl^/*Nestin*^cre^, *Atg7*^fl/fl^/*Pcp2*^Cre^, *FIP200*^fl/fl^/*Nestin*^cre^, and *ULK1/2*^*fl*/fl^/*Nesti*n^cre^ mice that specifically knock out autophagic genes in the nervous system have been generated, allowing the analysis of the roles of autophagy in neurodegeneration^9^. However, as a key autophagic component, the roles of VMP1 in central DAergic neurons have not been investigated in vivo. In the present study, we established *VMP1*^fl/fl^/*DAT*^CreERT2^ mouse model (referred as *VMP1*^cKO^) that postnatally knocks out autophagic gene *VMP1* in the cells expressing dopamine transporter (DAT) under the tamoxifen (TAM) treatment. We showed that the *VMP1*^cKO^ mice developed severe motor deficits accompanied by a substantial loss of mDAergic neurons and striatal axon terminals. Additionally, enlarged mDAergic axonal terminals that contain α-syn^+^ inclusions in the striatum were characterized in *VMP1*^cKO^ mice. Furthermore, the *VMP1*-deficient mDAergic neurons displayed damaged mitochondria, swollen ER, large vacuolar-like structures (LVSs), and accumulation of ubiquitin^+^ aggregates. Together, our studies provide strong evidence for the pathogenetic effects of *VMP1* deficiency on autophagy-mediated mDAergic neurodegeneration.

## Materials and methods

### Chemicals and antibodies

TAM, corn oil, and routinely used chemicals were purchased from Sigma-Aldrich (St. Louis, MO, USA). The information of antibodies used for WB and IFC staining was summarized in Table 1.

**Table 1:** Antibodies used in this study.

Target	Species	Application	Dilution	Company	Cat. number
TH	Mouse	WB, IFC	1:2000	Sigma	T1299
TH	Rabbit	WB, IFC	1:1000	Millipore	AB152
VMP1	Rabbit	WB	1:1000	Absin	abs126424
VMP1	Rabbit	IFC	1:300	Absin	abs126424
LC3β	Rabbit	WB	1:1000	Novus	NB100-2220
LC3β	Rabbit	IFC	1:400	Novus	NB100-2220
Beclin1	Rabbit	WB	1:1000	MBL	PD017
P62	Rabbit	WB	1:1000	Abcam	Ab109012
P62	Rabbit	IFC	1:400	Abcam	Ab109012
LAMP1	Rat	WB	1:1000	Abcam	Ab25245
MLKL (phospho S345)	Rabbit	IFC	1:400	Abcam	Ab196436
Ubiquitin	Mouse	IFC	1:200	SANTACRUZE	Sc8017
α-syn	Mouse	IFC	1:1000	BD	610786
GAPDH	Rabbit	WB	1:2000	CST	2118
Cleaved-caspase3 (Asp 175)	Rabbit	IFC	1:400	CST	9664
Phosphor-RIP3 (Thr231/Ser232)	Rabbit	IFC	1:400	CST	91702
SEC31A	Rabbit	IFC	1:400	CST	13466

### Generation of tissue-specific VMP1-deficient mice

The heterozygous mice *VMP1*^Flox/wt^ were generated by ViewSolid Biotech Co. Ltd (Beijing, China). Briefly, CRISPR technology was used to cut the intron 3 of the *VMP1* gene, and the donor vector having *loxP-*flanked exon 3 of *VMP1* was provided to have the insertion of *loxp* sites at mouse genomic *VMP1* DNA. According to the screen of cas9/gRNA activity and the target location, high-activity gRNAs (target DNA sequence: GAACAGAATTCTAGTCTCTGG; AAATATTGCTCTCCATTTGGG) were selected for microinjection into C57BL/6J fertilized eggs to construct conditional gene KO mice.

To achieve the mouse model of conditionally KO *VMP1* in the mDAergic neuronal system, *VMP1*^fl/fl^/*DAT*^CreERT2^ mice were produced by breeding mice carrying an inducible Cre recombinase under the *DAT* promoter with the heterozygous mice *VMP1*^Flox/wt^. The *DAT*^CreERT2^ mice were kindly gifted by the Günther Schütz group, which were generated by recombining a construct containing an improved Cre recombinase fused to a modified ligand-binding domain of the estrogen receptor into a bacterial artificial chromosome containing the gene encoding *DAT* ^10–12^.

All experimental mice were maintained under SPF conditions (temperature, 22 ± 2 °C; air exchange per 20 min; 12 h/12 h light/dark cycle) with free access to food and water. Animal care and procedures were carried out in accordance with the Laboratory Animal Care Guidelines approved by the Institutional Animal Care Committee at Dalian Medical University. The protocol was approved by the Institutional Animal Care Committee at Dalian Medical University.

### TAM treatment

TAM was dissolved in corn oil/ethanol (10:1) mixture at a final concentration of 10 mg/ml. Five to 6-week-old *VMP1*^fl/fl^ (*VMP1*^cWT^) and *VMP1*^fl/fl^/*DAT*^CreERT2^ (*VMP1*^cKO^) mice were both injected intraperitoneally with 1 mg TAM twice a day (total 2 mg/day) for 5 consecutive days, and then behavioral assessment was performed 2 weeks following the last injection of TAM. *VMP1*^cKO^ mice showed significant body loss at the 4th week following TAM injection, but the weight of *VMP1*^cWT^ mice was not altered. Both *VMP1*^cWT^ and *VMP1*^cKO^ mice were sacrificed at the 5th week following TAM injection. Some of the *VMP1*^cWT^ and *VMP1*^cKO^ mice were fasted for 6 h before sacrificed for IFC staining.

### Genotyping

*DAT*^CreERT2^ transgenic mice were identified by PCR screening (2 × EasyTaq PCR SuperMix, Transgen Biotech) of tail DNA using an antisense primer, CAG ACC AGG CCA GGT ATC TCT, and a sense primer, AGA ACC TGA TGG ACA TGT TCA GG, of which the transgene band size is 700 bp. The floxed *VMP1* knock-in mice were identified using AGCCGTCTCCTACTCCCTG and TGGTGATGGTTTTGTGCTTG. The PCR product size of the wild-type allele is 170 bp and the knock-in flox allele 204 bp.

### Locomotor activity

To measure the locomotor activity, *VMP1*^cWT^ and *VMP1*^cKO^ mice were placed into locomotor activity monitor instrument (25 × 25 × 30 cm, Med Associates Inc., St. Albans, USA) equipped with computer-controlled photocells. The activity was automatically recorded for 30 min, and total distance traveled, vertical time and counts, stereotypic time and counts calculated by the Med system. The behavioral assessment was performed between 13:00 and 16:00 on the 49th and 63rd postnatal day respectively.

### Rotarod test

As described previously^13^, mice were trained on the IITC Rotarod (IITC Life Science, Woodland Hills, CA) at 10 r/min, three times per day (at 1-h intervals) for 2 days, and were tested on the rotating rod with speed auto accelerating from 4 to 40 r/min over a period of 5 min. The length of time the mouse stayed on the rotating rod was recorded across three trials at 1-h intervals. The behavioral assessment was performed on the 45th, 47th, 49th, 59th, 61st, 63rd, and 65th postnatal day, respectively.

### Tail suspension test

Mice were suspended on a bar (50 cm above the floor) by the tail using a tape for 6 min. The cumulative immobility time during the last 4 min was recorded. The behavioral assessment was performed on the 60th postnatal day.

### Y-maze test

The Y-maze test instrument (Beijing Zhongshidichuang Science and Technology Development Col., Ltd, Beijing, China) was implemented in a white background with three arms (labeled as a–c arm) that extended from a central platform at a 120° angle. Each mouse was placed in the center of the Y-maze and was allowed to explore freely through the maze for 6 min. The sequence and the total number of arms entered were recorded using the observer. An arm entry was considered to be complete when the whole body of the mouse was completely placed within the arm. The behavioral assessment was performed on the 60th postnatal day.

### High-performance liquid chromatography (HPLC)

For the assessment of DA concentration, mice were sacrificed at the 5th week following TAM injection, and the striatum area was rapidly collected using a punch, weight and kept at −80 °C, which was performed for HPLC (EiCOM, HTEC-500, USA) as described in detail previously^14^.

### Western blot (WB)

Mice were sacrificed at the 5th week following TAM injection, and the tissues were dissected rapidly on ice and homogenized in cold RIPA buffer (Beyotime Biotechnology, Shanghai, China) containing protease and phosphatase inhibitor cocktails (Sigma-Aldrich, St. Louis, MO, USA) and then lysed for 30 min on ice. The protein concentration in the supernatant was determined using protein assay kits (TaKaRa, Shiga, Japan). Forty micrograms of protein were loaded and separated by sodium dodecyl sulfate/polyacrylamide gel electrophoresis and then transferred to polyvinylidene fluoride membranes (Millipore, Bedford, MA, USA). After blocking, membranes were incubated with appropriate primary antibodies (Table 1) at 4 °C overnight, followed by 1-h incubation at room temperature with a peroxidase-conjugated secondary antibody. Finally, the membrane was incubated with enhanced chemiluminescence (Millipore, Bedford, MA, USA), and the target protein bands were quantified using the FluorChem Q system (ProteinSimple, California, USA).

### IFC staining

For histological analysis, mice were anesthetized with ketamine at the 5th week following TAM injection and perfused transcardially with 4% paraformaldehyde (PFA)^15^. After dehydrated in 30% sucrose, the brain tissues were cut into 40 μm coronal sections using Leica cryostat (CM-1950S, Leica, Germany). They were incubated with blocking buffer (10% normal goat serum, 1% bovine serum albumin, 0.3% Triton X-100, PBS solution) overnight at 4 °C and were then incubated with the primary antibodies overnight at 4 °C (Table 1). For Ub staining, the sections were performed antigen repair by using citrate buffer (pH 6.0). The stained sections were imaged using a laser scanning confocal microscope (A1 confocal, Nikon instruments (Shanghai) Co., Ltd). The paired images in the figures were collected at the same gain and offset settings.

### Transmission electron microscope (TEM) analysis

Mice were sacrificed at the 5th week following TAM injection, and the midbrain area was collected rapidly on ice within 3 min into a fixative solution containing 2.5% glutaraldehyde (Servicebio, Wuhan, China) and then fixed at room temperature for 2 h followed by transferring to 4 degrees for storage. The tissues were washed three times in PBS (pH 7.4) before post-fixing in 1% osmium acid (diluted with 0.1 M PBS solution) at room temperature for 2 h and were successively dehydrated. After a series of embedding steps, the tissues were cut into 80 nm sections using the Leica ultrathin microtome (Leica UC7, Leica, Germany) and stained with 2% uranyl acetate saturated alcohol solution and lead citrate solution, respectively. The stained sections were imaged using a TEM (HITACHI, HT7700).

### Image analysis

TH^+^ cells in SNc and VTA were calculated in every three sections from Bregma −2.70 to −3.88 mm at a magnification of ×10 by an observer who was blind to the genotype and grouping, and the data were collected from 8 to 10 slice per animal^14^. The outline of SNc and VTA was determined according to anatomical landmarks^16^. The analysis of IFC staining on the number of puncta, axon density, mean number of enlarged axon terminals were quantified using the Image J software, and the data were collected from 3 to 4 slices per animal.

### Statistical analysis

Data were expressed as means ± SEM and were analyzed using GraphPad Prism software (Version 7.0). Protein bands were quantified using the FluorChem Q system (ProteinSimple, California, USA). Two-way ANOVA followed by Sidak’s multiple comparisons test was used for analyses across multiple groups, with Student’s *t*-test used to determine significant differences between two groups. Data were presented as means ± SEM, and *p* < 0.05 was considered significant. All experiments were repeated at least three times and sample sizes were estimated from pilot experiments. No statistical methods were used to predetermine sample size, but our sample sizes are similar to those reported in previous publications.

## Results

### VMP1^cKO^ mice develop severe motor deficits

**Fig. 1:**
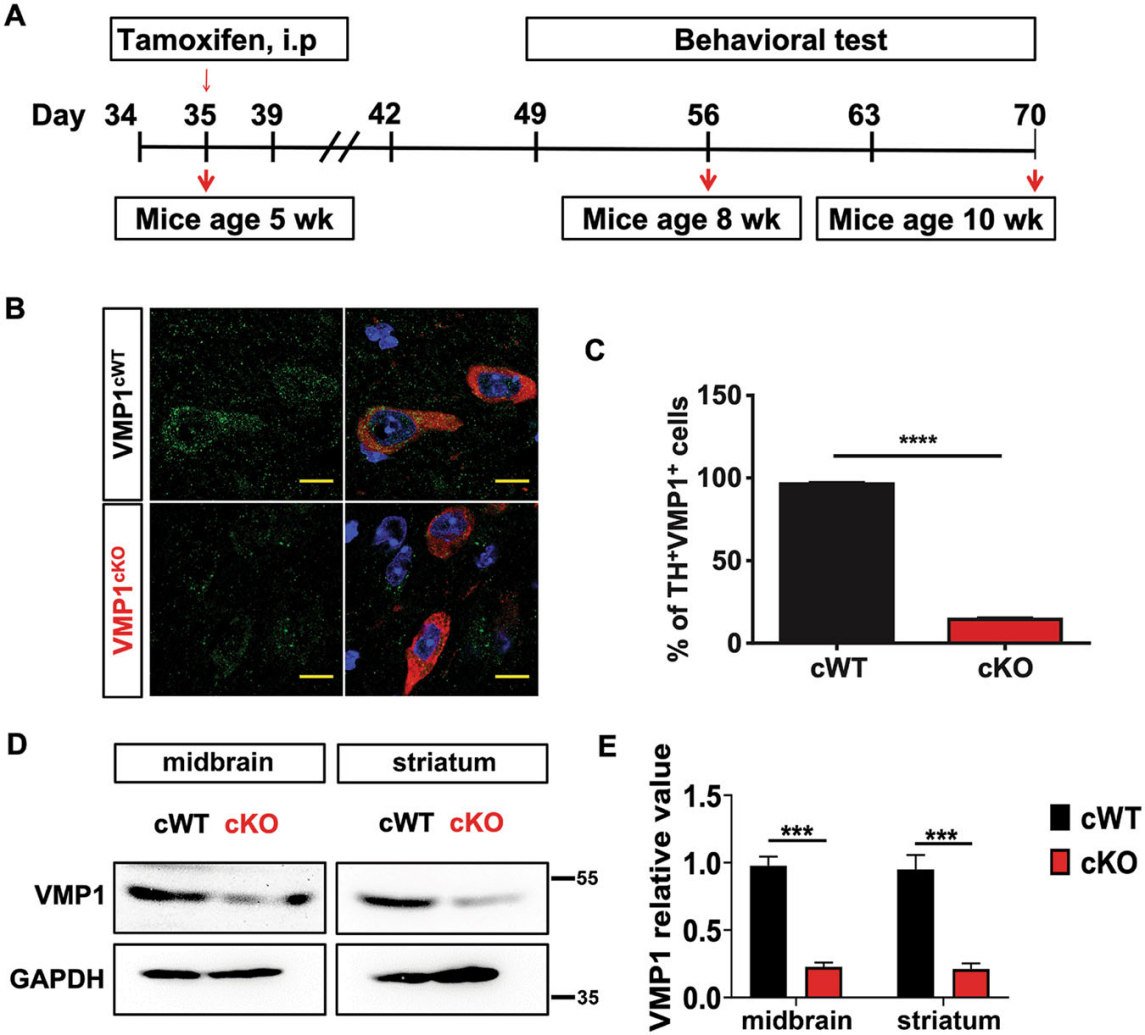
Conditional knockout of *VMP1* in the mDAergic neurons. **A** A simplified scheme of the experimental timeline for the administration of TAM to both *VMP1*^cWT^ and *VMP1*^cKO^ mice. **B** IFC analysis for VMP1 expression in the SNc was performed by using an antibody against VMP1 (Green) together with TH (Red) in *VMP1*^cWT^ and *VMP1*^cKO^ mice. The nuclei were labeled with DAPI (Blue). Scale bar, 10 μm. **C** The percentage of TH^+^ and VMP1^+^ neurons in the SNc of *VMP1*^cWT^ and *VMP1*^cKO^ mice (*N* = 3 mice per genotype). **D** WB analysis for the VMP1 expression levels in the midbrain and striatum. **E** Quantification of WB analysis of (**D**) (*N* = 3 mice per genotype). **C** Was analyzed by using Student’s *t*-test. **E** Was analyzed by using two-way ANOVA followed by Sidak’s multiple comparisons test. Data were represented as mean ± SEM. ***p < 0.001, ****p < 0.0001.

**Fig. 2:**
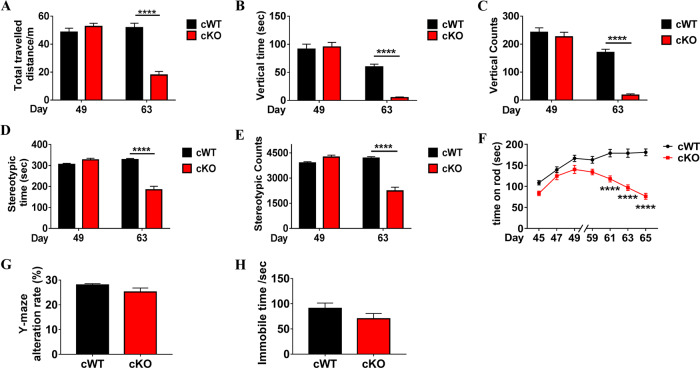
Motor deficits in *VMP1*^cKO^ mice. Total traveled distance (**A**), vertical time (**B**), vertical counts (**C**), stereotypic time (**D**), and stereotypic counts (**E**) were presented, respectively (*N* = 24 mice per genotype). **F** The latency to fall from rotarod was recorded from *VMP1*^cWT^ and *VMP1*^cKO^ mice from the 45th to 65th postnatal day (*N* = 23–24 mice per genotype). **G** The mice entered a, b, or c arm were recorded, and the Y-maze alteration rate was calculated (*N* = 16–20 mice per genotype). **H** The immobile time was recorded in the tail suspension test (*N* = 9–12 mice per genotype). **A–F** were analyzed by using two-way ANOVA followed by Sidak’s multiple comparisons test. **G**, **H** were analyzed by using Student’s *t*-test. Data were represented as mean ± SEM. ****p < 0.0001.

VMP1 is one of the core components of ATG machinery^2,17^. To determine its role in mammalian mDAergic neurons, we first established the mouse model with mDAergic neuron-specific deletion of *VMP1* (the construction for TAM-inducible *VMP1*-deficient mice as shown in Supplementary Fig. 1A). At the 5 weeks old, *VMP1*^cWT^and *VMP1*^cKO^ mice were both injected intraperitoneally with TAM (Fig. 1A). We recorded the body weight and physical condition of the mice and found *VMP1*^cKO^ mice showing significant body loss at the 4th week following injection (Supplementary Fig. 1B, C). Mice were then sacrificed at the 70th postnatal day. The VMP1 expression profile in the SNc was firstly detected using immunofluorescence (IFC) staining. As expected, the expression level of VMP1 in the SNc mDAergic neurons (tyrosine hydroxy (TH)-labeled) of *VMP1*^cKO^ mice was dramatically decreased (Fig. 1B, C), indicating the success of *VMP1* depletion. Moreover, the biochemical analysis of midbrain and striatal homogenates from *VMP1*^cKO^ and *VMP1*^cWT^ mice by WB confirmed *VMP1* depletion in the mDAergic neurons (Fig. 1D, E). To assess the impact of *VMP1* depletion on motor activity, the open-field test was performed on the 49th and 63rd postnatal day, respectively. We found that the total distance traveled, vertical movement (vertical time and counts), and stereotypic movement (stereotypic time and counts) were significantly decreased in *VMP1*^cKO^ mice at the 63rd postnatal day compared to the age-matched *VMP1*^cWT^ mice (Fig. 2A–E), demonstrating that *VMP1*^cKO^ mice developed progressive motor deficits. In addition, video recording at the 63rd postnatal day showed that *VMP1*^cKO^ mice displayed an unbalanced, trembling walking pattern, which was not observed in *VMP1*^cWT^ mice (Supplementary Videos 1 and 2). Furthermore, the rotarod task showed that *VMP1*^cKO^ mice stayed less time on the rotating rod (Fig. 2F), suggesting a progressive pattern of impaired motor coordination in *VMP1*^cKO^ mice. In addition, we performed a Y-maze test and tail suspension test at the 60th postnatal day and found no significant difference, suggesting no cognitive impairment in *VMP1*^cKO^ mice (Fig. 2G). Moreover, the immobility time was comparable between *VMP1*^cKO^ and *VMP1*^cWT^ mice in the tail suspension test (Fig. 2H)^18,19^.

### VMP1^cKO^ mice display a profound mDAergic neuronal loss and enlarged axonal terminals

**Fig. 3:**
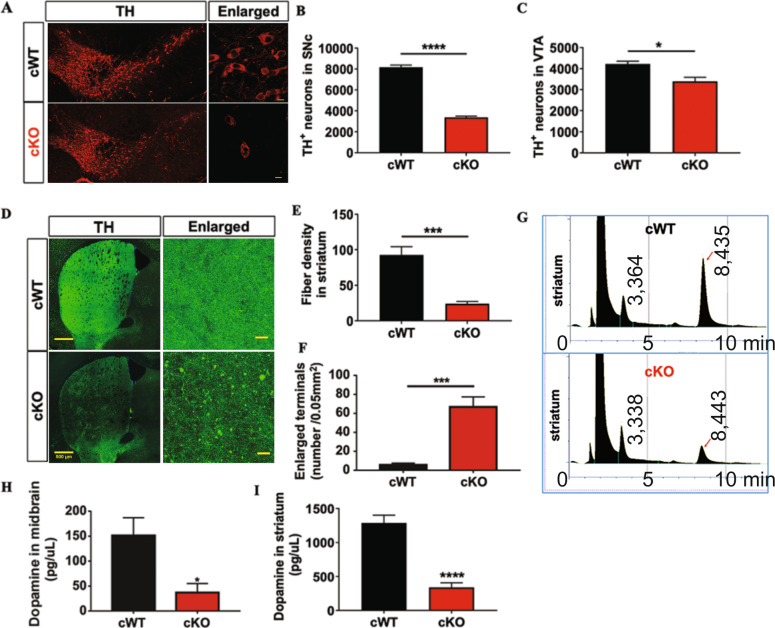
Neuropathology examination and DA measurement in the SNc-striatal pathway of *VMP1*^cKO^ mice. **A** The IFC staining for the mDAergic neurons was performed by using the antibody against TH (red) in *VMP1*^cWT^ and *VMP1*^cKO^ mice. Scale bar, 100 μm. Scale bar of the high-magnification images, 15 μm. Quantitation of TH^+^ cells in the SNc (**B**) and VTA (**C**) from *VMP1*^cWT^ and *VMP1*^cKO^ mice, respectively (*N* = 3 mice per genotype). **D** IFC staining for the axonal density of the mDAergic neurons was performed by using the antibody against TH (green) in *VMP1*^cWT^ and *VMP1*^cKO^ mice. The nuclei were labeled with DAPI (blue). Scale bar, 500 μm. Scale bar of the high-magnification images, 25 μm. **E** Quantitation of TH^+^ fiber density in the striatum from *VMP1*^cWT^ and *VMP1*^cKO^ mice (*N* = 3 mice per genotype). **F** Mean number of the enlarged axon terminals (area >0.5 μm^2^) per 0.05 mm^2^ perspective (*N* = 3 mice per genotype). **G** The peak time of DA flux. DA concentration was assessed by HPLC in the midbrain (**H**) and striatum (**I**), respectively (*N* = 7 mice per genotype). Data were analyzed by using Student’s *t*-test and were represented as mean ± SEM. *p < 0.05, ***p < 0.001, ****p < 0.0001.

To analyze the correlation between motor deficit and loss of mDAergic neurons, IFC staining of TH, a representative marker for DAergic neurons, was performed for the quantification of mDAergic neurons. The number of mDAergic neurons in the SNc and VTA from the 10-week-old *VMP1*^cKO^ mice was markedly decreased by 59% and 20%, respectively, compared to the age-matched *VMP1*^cWT^ mice (Fig. 3A–C). Furthermore, the fiber density of mDAergic axonal terminals sharply declined along with the presence of the massive enlargements (Fig. 3D–F), indicating that *VMP1* deficiency in the mDAergic neurons leads to neuronal damage in both soma and axons. Dopamine (DA), the distinct catecholamine neurotransmitter synthesized by DAergic neurons, was measured by HPLC (Fig. 3G–I)^20,21^; TH, a key enzyme for DA synthesis, was examined by WB (Supplementary Fig. 2). The mean concentration of DA was notably reduced from 151.6 pg/μL in the midbrain of *VMP1*^cWT^ mice to 36.85 pg/μL in that of *VMP1*^cKO^ mice (Fig. 3H), and from 1287 pg/μL in the striatum of *VMP1*^cWT^ mice to 340 pg/μL in that of *VMP1*^cKO^ mice (Fig. 3I). Correspondingly, the TH protein levels in the midbrain and striatum were both decreased by 52% and 58% in *VMP1*^cKO^ mice compared with *VMP1*^cWT^ (Supplementary Fig. 2). Together, our data reveal the severe dysfunction of mDAergic nervous system in *VMP1*^cKO^ mice.

### Necroptosis in the mDAergic neurons of VMP1^cKO^ mice

**Fig. 4:**
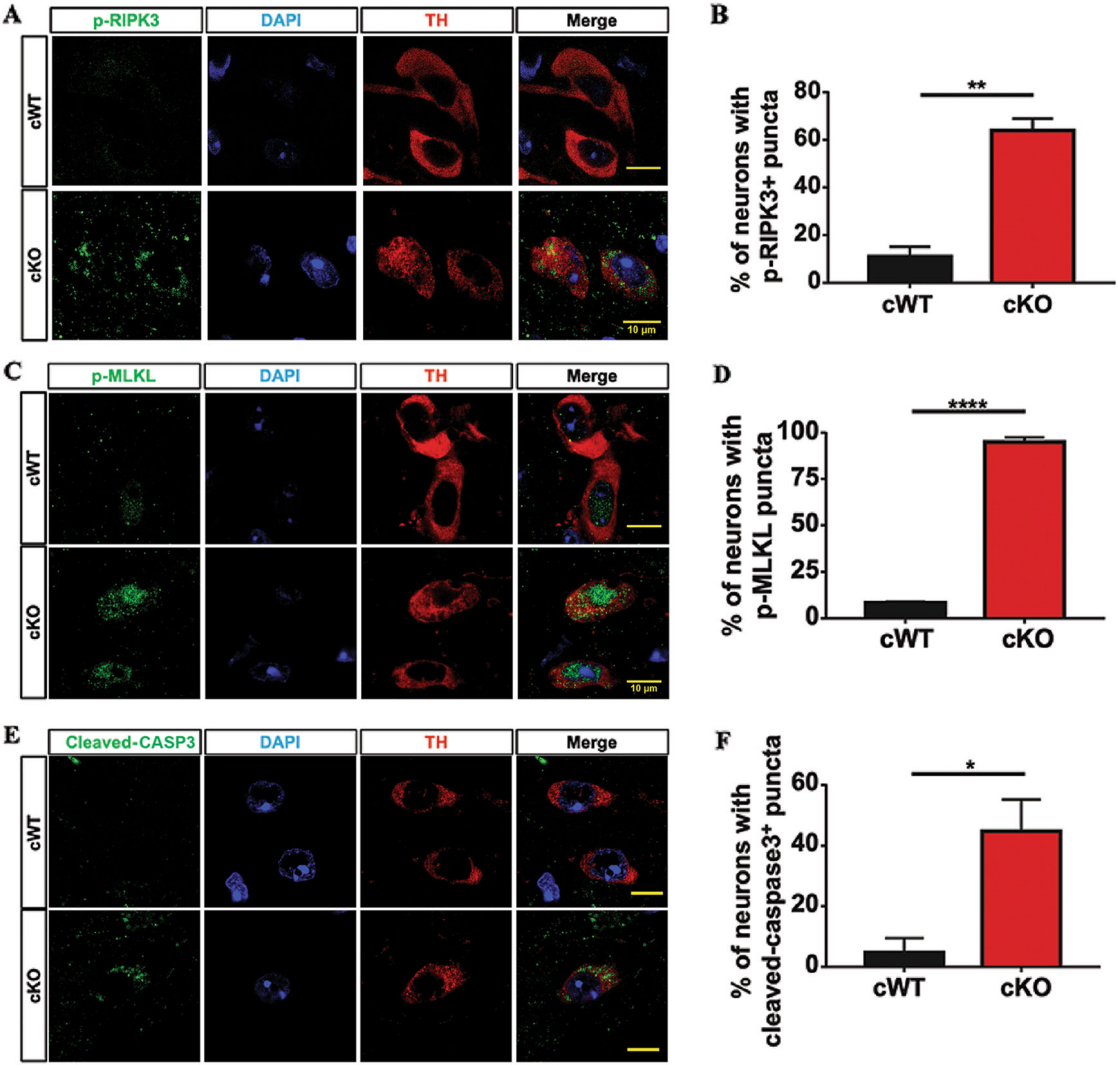
Necroptosis detection in the mDAergic neurons of *VMP1*^cKO^ mice. Double-label immunofluorescence of p-RIP3 (Thr231/Ser232) (green) and TH (red) in the mDAergic neurons of *VMP1*^cKO^ and *VMP1*^cWT^ mice. Scale bar, 10 μm. **B** The proportion of TH^+^ neurons with p-RIPK3 puncta was quantified (*N* = 3 mice per genotype). **C** Double-label IFC staining of p-MLKL (phospho S345) (green) and TH (red) in the SNc of *VMP1*^cKO^ and *VMP1*^cWT^ mice. Scale bar, 10 μm. **D** The proportion of TH^+^ neurons with p-MLKL puncta was quantified (*N* = 3 mice per genotype). **E** Double-label immunofluorescence of cleaved-caspase3 (Asp175) (green) and TH (red) in the SNc of *VMP1*^cKO^ and *VMP1*^cWT^ mice. Scale bar, 10 μm. **F** The proportion of TH^+^ neurons with cleaved-caspase3 puncta was quantified (*N* = 3 mice per genotype). Data were analyzed by using Student’s *t*-test and were represented as mean ± SEM. *p < 0.05, **p < 0.01, ****p < 0.0001.

Since *VMP1*^cKO^ mice displayed a profound mDAergic neuronal loss, we tried to further elucidate the mechanism underlying cell death^22^. Necroptosis has been described as a highly regulated form of necrosis and considered to be a highly pro-inflammatory mode of cell death^23–25^. When necroptosis is induced, receptor-interacting protein kinase-3 (RIPK3) is activated by phosphorylation, and the activated RIP3 phosphorylates mixed lineage kinase like (MLKL) at the threonine 349, serine 345, and serine 347 residues^26,27^. Based on these previous studies, we thereby examined whether the mDAergic neuronal loss upon *VMP1* deficiency is associated with necroptosis. Using the antibody against phospho-RIPK3 (Thr231/Ser232) and MLKL (phospho S345), we performed IFC staining and found that in the mDAergic neurons of *VMP1*^cKO^ mice, p-RIPK3 was dramatically concentrated and formed discrete puncta at the cytoplasm (Fig. 4A, B). Correspondingly, p-MLKL was significantly accumulated and formed large puncta at the nucleus and cytoplasm of the mDAergic neurons (Fig. 4C, D), indicating necroptosis is induced in the mDAergic neurons due to *VMP1* deficiency. Next, we examined the activation of caspase-3 in the midbrain region, showing that the mDAergic neurons in *VMP1*^cKO^ mice displayed concentrated cleaved-caspase3 (Asp175) puncta, while the caspase-3 activation was barely observed in *VMP1*^cWT^ mice (Fig. 4E, F). These results indicate that besides necroptosis, apoptosis might be also involved in the neuronal death caused by *VMP1* deficiency.

### Disrupted autophagic flux in the mDAergic neurons of VMP1^cKO^ mice

**Fig. 5:**
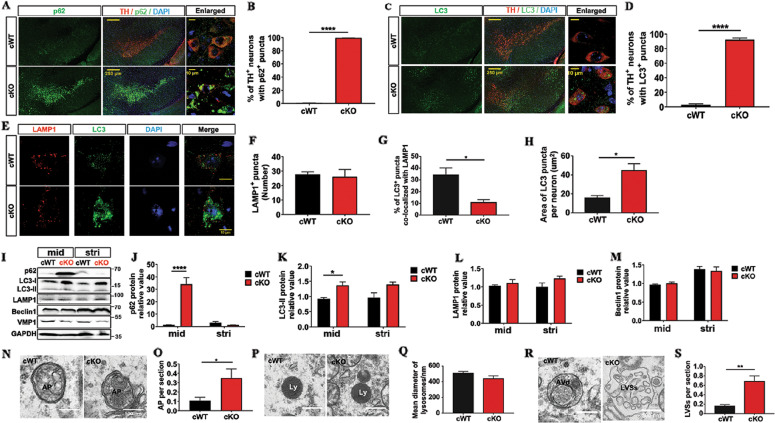
Autophagic flux disruption in *VMP1*^cKO^ mice. **A** IFC staining for p62 in the SNc was performed by using the antibody against p62 (green) together with TH (red). The nuclei were labeled with DAPI (Blue). Scale bar, 250 μm. Scale bar of the high-magnification images, 10 μm. **B** The proportion of TH^+^ cells with p62 puncta (>1 μm^2^) was quantified (*N* = 3 mice per genotype). **C** IFC staining for LC3 in the SNc was performed by using the antibody against LC3 (Green) together with TH (Red). The nuclei were labeled with DAPI (Blue). Scale bar, 250 μm. Scale bar of the high-magnification images, 10 μm. **D** The proportion of TH^+^ cells with LC3 puncta (>0.5 μm^2^) was quantified (*N* = 3 mice per genotype). **E** Double-label immunofluorescence of LC3 (green) and LAMP1 (red) in the mDAergic neurons. Scale bar, 10 μm. **F** The number of LAMP1^+^ puncta (>0.1 μm^2^) per TH^+^ neuron (*N* = 3 mice per genotype). **G** Quantification of the area of LC3^+^ puncta per TH^+^ neuron (*N* = 3 mice per genotype). **H** The proportion of LC3^+^ puncta co**-**localized with LAMP1 was quantified (*N* = 3 mice per genotype). **I** WB analysis for p62, LC3, LAMP1 and Beclin1 protein expression levels in the midbrain and striatum. GAPDH as an internal reference. **J–M** Quantification of p62, LC3II, LAMP1 and Beclin1 relative to GAPDH was shown, respectively (*N* = 3 mice per genotype). The representative TEM images of the AP (**N**), Ly (**P**), as well as AVd and LVSs (**R**) in cells. Scale bar, 0.5 μm. The quantification of the number of AP per cell section (**O**), the diameters of Ly (**Q**), and the number of LVSs per cell section (**S**) was shown. **B**, **D**, **F**, **G**, **H**, **O**, **Q**, **S** were analyzed by using Student’s *t*-test. **J–M** were analyzed by using two-way ANOVA followed by Sidak’s multiple comparisons test. Data were represented as mean ± SEM. *p < 0.05, **p < 0.01, ****p < 0.0001. mid midbrain, stri striatum, Ly lysosome, AP autophagosome, AVd degradative autophagic vacuoles, LVSs large vacuolar-like structures.

A constitutive level of autophagy is important for maintaining cellular homeostasis under normal conditions. Thus, we focused on whether *VMP1* deficiency disrupts the autophagic flux in the mDAergic neurons. A large number of p62 puncta was accumulated in the soma of the mDAergic neurons of *VMP1*^cKO^mice (Fig. 5A, B). Similarly, large LC3^+^ puncta were concentrated in the cell body of the mDAergic neurons in *VMP1*^cKO^ mice but not in *VMP1*^cWT^ mice (Fig. 5C, D), suggesting that LC3-labeled autophagic luncta accumulate in the mDAergic neurons resulting from *VMP1* deficiency. Double-staining results revealed that LC3 puncta were normally co-localized with lysosomal-associated membrane protein 1 (LAMP1)-labeled lysosomes in the mDAergic neurons of fasted *VMP1*^cWT^ mice (Fig. 5E–H). However, such co-localization was disrupted in matched fasted *VMP1*^cKO^ mice (Fig. 5E–H), showing most LC3 signals failed to merge with LAMP1 signals. Immunoblotting results also confirmed that p62 and LC3 levels were both increased in the midbrain of *VMP1*^cKO^ mice compared with *VMP1*^cWT^ mice (Fig. 5I–K). Neither LAMP1 nor Beclin1 protein expression level was altered when compared between the midbrain of *VMP1*^cKO^ mice and *VMP1*^cWT^ mice (Fig. 5L, M). Furthermore, TEM analysis showed an increase in the number of autophagosomes in *VMP1*^cKO^ mDAergic neurons (Fig. 5N, O), indicating that autophagic flux at the fusion stage is impaired in the *VMP1*-deficient mDAergic neurons, in accordance with the previous report^4^. Although the fusion of the autophagosome with lysosome was blocked upon *VMP1* depletion (Fig. 5E–H), the morphological alteration of *VMP1* deficiency on the lysosome was not identified (Fig. 5P, Q). Degradative autophagic vacuoles (AVd) (including autolysosomes and amphisomes) typically have monolayer membrane, and usually contain electron-dense cytoplasmic material and/or organelles at various stages of degradation^28^. We found a substantial number of AVd in the mDAergic neurons from the *VMP1*^cWT^ mice (Fig. 5R). However, in *VMP1*^cKO^ mice, we hardly found AVd but saw abundant LVSs in irregular morphology with enlarged diameters (>500 nm) and clear content of lumenal material (Fig. 5S), indicating the disruption of degradation steps.

### Defects of mitochondria in the mDAergic neurons of VMP1^cKO^ mice

**Fig. 6:**
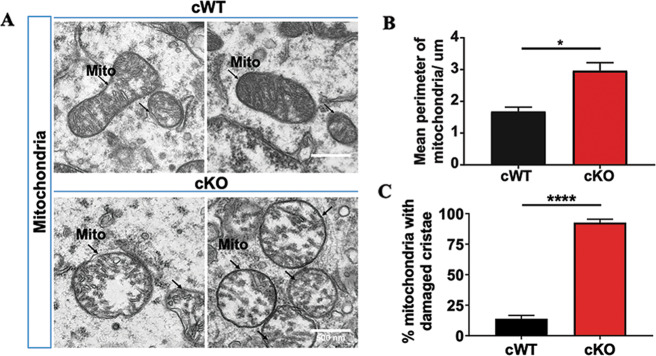
Mitochondrial defects in the mDAergic neurons of *VMP1*^cKO^ mice. **A** The representative TEM images of the observed mitochondria. Scale bar, 0.5 μm. **B** Quantification of the mean perimeter of mitochondria (*N* = 3 mice per genotype). **C** The proportion of mitochondria with damaged cristae was quantified (*N* = 351 mitochondria collectively counted from 3 *VMP1*^cWT^ mice, *N* = 291 mitochondria collectively counted from 3 *VMP1*^cKO^ mice). Data were analyzed by using Student’s *t*-test and were represented as mean ± SEM. *p < 0.05, ****p < 0.0001. Mito mitochondria.

Next, we determined the impact of *VMP1* deficiency on the organelles of the mDAergic neurons. TEM analysis was also performed to assess the mitochondrial morphology. A lot of spherical mitochondria were found in the mDAergic neurons of *VMP1*^cKO^ mice but not in *VMP1*^cWT^ mice (Fig. 6A). Furthermore, compared to the *VMP1*^cWT^ mice, the mean perimeter of the mitochondria was dramatically increased, an indication of swollen, in the mDAergic neurons of *VMP1*^cKO^ mice (Fig. 6B). Additionally, more than 90% of the mitochondria cristae in the mDAergic neurons of *VMP1*^cKO^ mice were reduced, broken or even disappeared, indicating that mitochondria are damaged upon *VMP1* deficiency in the mDAergic neurons (Fig. 6C).

### Altered rough ER structure in the mDAergic neurons of VMP1^cKO^ mice

**Fig. 7:**
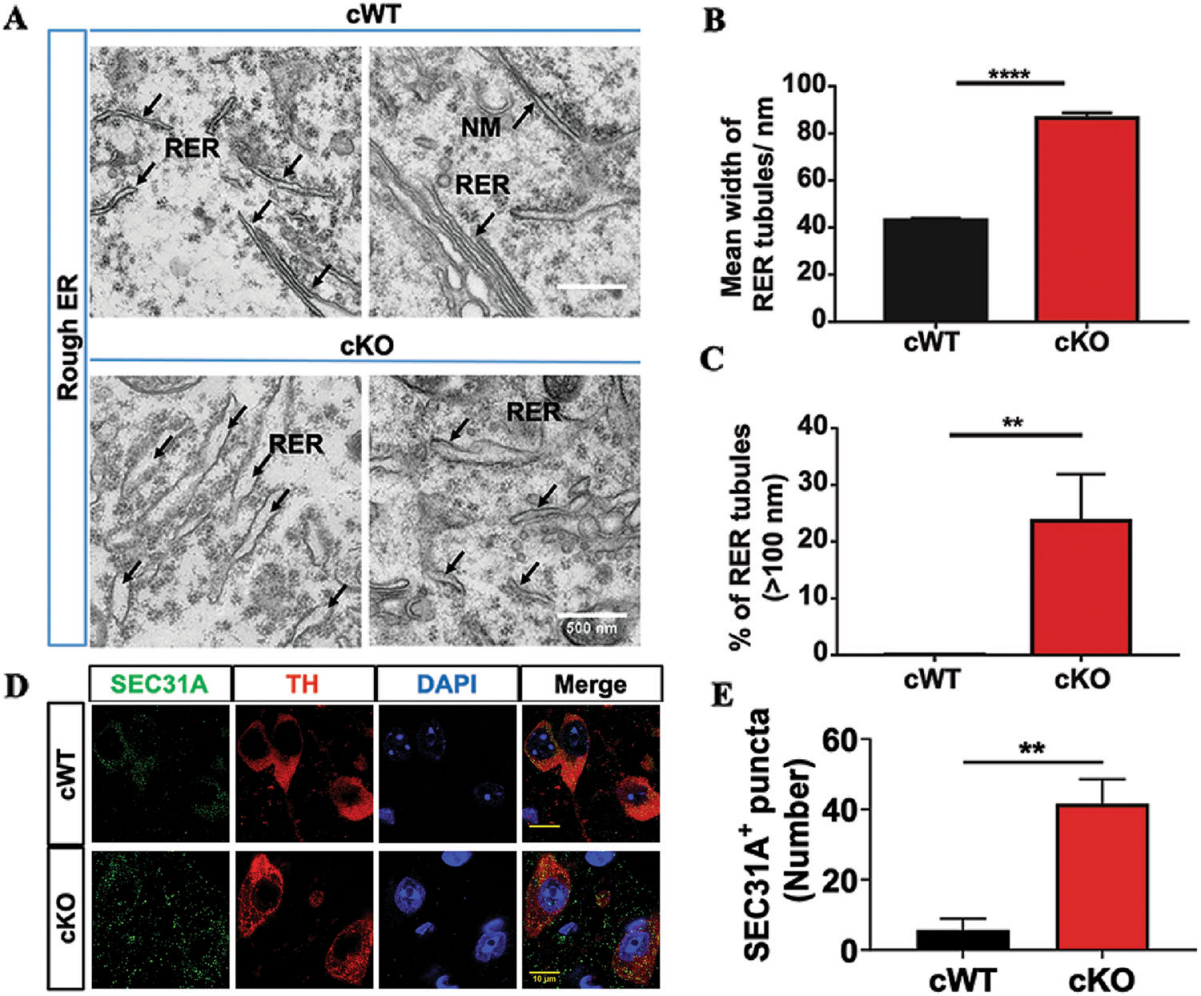
The morphological alterations of RER in the mDAergic neurons of *VMP1*^cKO^ mice. **A** The representative TEM images of the observed RER. Scale bar, 0.5 μm. **B** The mean width of RER tubules was shown (*N* = 487 RER collectively counted from 3 *VMP1*^cWT^ mice, *N* = 500 RER collectively counted from 3 *VMP1*^cKO^ mice). **C** The proportion of RER tubules (>100 nm) was quantified from (**B**). **D** Double-label immunofluorescence of SEC31A (green) and TH (red) in the mDAergic neurons. The nuclei were labeled with DAPI (blue). Scale bar, 10 μm. **E** Quantification of the number of SEC31A^+^ puncta (>0.1 μm^2^) per TH^+^ neuron (*N* = 3 mice per genotype). Data were analyzed by using Student’s *t*-test and were represented as mean ± SEM. **p < 0.01, ****p < 0.0001. RER rough endoplasmic reticulum, NM nuclear membrane, RER rough ER.

We next analyzed the rough ER (RER) structure and found a significant change in the morphology of RER in *VMP1*^cKO^ mice compared with *VMP1*^cWT^ mice (Fig. 7A). The mean width of RER tubules doubled in the mDAergic neurons of *VMP1*^cKO^ mice (83.6 nm) compared to that in *VMP1*^cWT^ mice (40.8 nm) (Fig. 7B), and the proportion of RER tubules (>100 nm) was increased from 0.05% in the mDAergic neurons of *VMP1*^cWT^ mice to 16.65% in *VMP1*^cKO^ mice (Fig. 7C). These results indicate that the mDAergic neurons suffer from severe RER damage because of *VMP1* deficiency. It is known that SEC31A (SEC31 Homolog A, COPII coat complex component) is involved in vesicle budding from the ER and ER–Golgi transport^29^. We found that SEC31A was accumulated to form large, discrete puncta in the mDAergic neurons of *VMP1*^cKO^ mice (Fig. 7D, E). Correspondingly, the mRNA levels of SEC16A (SEC16 Homolog A, ER export factor from ER to Golgi), SEC31A, and SEC31B (SEC31 Homolog B, COPII coat complex component) were significantly upregulated in *VMP1*^cKO^ mice (Supplementary Fig. 3). These results suggest that vesicle budding of ER and ER–Golgi transport are abnormal upon *VMP1* deficiency.

### Accumulation of α-syn inclusion and abnormal protein aggregation in the nigrostriatal projection of VMP1^cKO^ mice

**Fig. 8:**
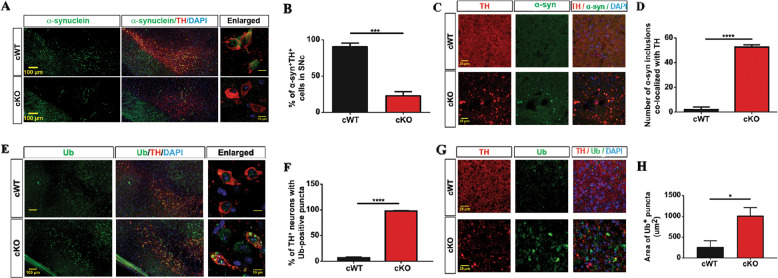
Accumulation of the α-syn inclusions at the striatal axonal terminals in *VMP1*^cKO^ mice. **A** IFC analysis for α-syn expression in the SNc was performed by using the antibody against α-syn (green) together with TH (red). The nuclei were labeled with DAPI (blue). Scale bar, 100 μm. Scale bar of the high-magnification images, 10 μm. **B** The proportion of α-syn^+^-TH^+^ cells in the SNc was quantified (*N* = 3 mice per genotype). **C** IFC analysis for α-syn expression in the striatum was performed by using the antibody against α-syn (green) together with TH (red). The nuclei were labeled with DAPI (blue). Scale bar, 25 μm. **D** Mean number of α-syn inclusions co-localized with TH in the striatum (*N* = 3 mice per genotype). **E** IFC analysis for Ub^+^ protein expression in the SNc by using the antibody against Ub (green) together with TH (red). The nuclei were labeled with DAPI (blue). Scale bar, 100 μm. Scale bar of the high-magnification images, 10 μm. **F** The proportion of TH^+^ neurons in the SNc with Ub^+^ puncta was quantified (*N* = 3 mice per genotype). **G** IFC staining for Ub in the striatum by co-staining Ub (green) together with TH (red). The nuclei were labeled with DAPI (blue). Scale bar, 25 μm. **H** Total area of Ub^+^ puncta (>0.5 μm^2^) per 0.05 mm^2^ in the striatum was quantified (*N* = 3 mice per genotype). Data were analyzed by using Student’s *t*-test and were represented as mean ± SEM. *p < 0.05, ***p < 0.001, ****p < 0.0001.

To further clarify the potential role of VMP1 in the mDAergic neuron degeneration, we examined the aggregation of endogenous α-syn by IFC staining. We found that α-syn was diffusely present in the soma of the mDAergic neurons from the *VMP1*^cWT^ mice (Fig. 8A), which is consistent with other reports that endogenous α-syn was presented in the SNc and VTA of C57Bl/6J mice^30–32^. However, in our age-matched *VMP1*^cKO^ mice, the endogenous α-syn protein level in the soma was sharply reduced and no obvious presence of inclusions (Fig. 8A, B). Unexpectedly, we found that α-syn inclusions were highly accumulated at the enlarged axon terminals in the striatum of *VMP1*^cKO^ mice (Fig. 8C, D), revealing the profound impact of VMP1 on the α-syn transportation at the axonal terminals of the mDAergic neurons. Our data thus provide evidence for VMP1’s role in regulating α-syn axonal transportation.

We next examined protein aggregation in the midbrain and striatum using the antibody against ubiquitin, a classical marker of misfolded proteins. We found that large ubiquitin^+^ puncta were accumulated in the cytoplasm of mDAergic neurons of *VMP1*^cKO^ mice, whereas such aggregations were not observed in *VMP1*^cWT^ mice (Fig. 8E, F). Similarly, we also found large ubiquitin^+^ puncta concentrated in the striatum, but they were not significantly co-localized with axon terminal enlargements (Fig. 8G, H). These findings suggest the accumulation of misfolded proteins in both the midbrain and striatum, resulting from *VMP1* deficiency.

## Discussion

Increasing evidence indicates that VMP1 may act as a platform promoting the upstream autophagic events^2,33,34^. Extensive in vitro studies have demonstrated that the *VMP1* deficiency blocks autophagic flux by suppressing the fusion of autophagosome with lysosome^4^. However, due to the absence of a physiological cellular context, it is unclear how these changes affect the specific neurons in vivo. Here, we thereby reported for the first time that conditional KO of *VMP1* in the fully differentiated mDAergic neurons caused significant motor deficiency, such as imbalance, tremor, and ataxic walking. Meanwhile, these movement disorders were concomitant with the loss of mDAergic neurons and fibers, as well as the swollen terminals. Interestingly, the enlarged axonal puncta were highly colocalized with α-syn aggregates, suggesting the dysfunction of VMP1 may contribute significantly to α-syn-induced pathogenesis in the mDAergic neurons. At the molecular level, a proportion of the accumulated LC3II^+^ and p62^+^ aggregates was presented in the *VMP1*-deficient mDAergic neurons. LC3II, a standard marker for autophagosomes, is specifically associated with autophagosomes and autolysomes; p62, a classical receptor of autophagy participates in the cytoplasmic cargos’ transportation, and itself is autophagy substrate. Thus, the appearance of such LC3II^+^ and p62^+^ aggregates may indicate that *VMP1* deficiency severely impairs the autophagy activity/flux in the mDAergic neurons^35^.

Autophagy is a key factor for keeping the homeostasis of cells by removing misfolded proteins or toxic components. VMP1 is an important component in the autophagic system in regulating interactions between the ER and the autophagic-isolation membrane. The conventional KO mice of *VMP1* are embryonic lethal^36^. Therefore, for further mechanistic investigation, we have constructed conditionally KO mice that using TAM to induce postnatal deletion of the *VMP1* gene specifically in the cells expressing DAT. Our *VMP1*^cKO^ mice have about 5 weeks of survival after TAM treatment and they suffer from significant body loss at the 4th week and then die within one week with a profound mDAergic neuronal degeneration. Compared with Atg7^DatCre^ mice that displayed a substantial loss of the mDAergic neurons at one year of age^37^, the *VMP1*^cKO^ mice have a much more severe phenotype, nearly 60% of the mDAergic neuronal loss at 4th week following the TAM treatment, indicating that VMP1 is essential for the survival of the mDAergic neurons. As expected, we found massive Ub^+^ proteins accumulation and the LVSs formation in the cytoplasm of the mDAergic neurons of *VMP1*^cKO^ mice, suggesting the severe impairment of protein degradation functions in the autophagic system because of *VMP1* deficiency. It is likely the components of LVSs are autophagosome-related vacuoles. Thus, the accumulation of LVSs in *VMP1*^cKO^ mDAergic neurons may indicate the dysfunction of local membrane trafficking and turnover. Furthermore, we have documented severely disrupted mitochondria in the mDAergic neurons of *VMP1*^cKO^ mice. It is known that the removal of damaged mitochondria through autophagy, termed mitophagy, is indispensable for maintaining proper cellular functions^38^. Whereas, in our *VMP1*^cKO^ mice autophagic flux was dramatically disrupted by the failure of the lysosome to fuse with autophagosome, resulting in the damaged mitochondria highly accumulated. These retained dysfunctional mitochondria are mainly characterized by the hallmarks of decreased ΔΨ_m_ and lower levels of OPA1^39^. Mitochondria fusion is thought to be a less-selective process, meaning that the probability of mitochondria with lower ΔΨ_m_ to fuse is high^40^. Such aberrant fusion may largely affect the mitochondrial fission step and significantly contribute to the observed swelling phenotype. In addition, we have observed the extensive swollen ER in the mDAergic neurons of *VMP1*^cKO^ mice, suggesting that the failure in handling of misfolded proteins is associated with the damage of several organelles. On the other hand, VMP1 is ubiquitously expressed in an extensive network of membranes, and ER establishes membrane contact sites with most organelles by signaling events and molecular trafficking. Previous studies suggest that VMP1 may be involved in a tight spatial and temporal regulations of PtdIns3P signaling and membrane source recruitment by restricting the ER–mitochondria contact sites^4^. Depletion of *VMP1* in HeLa and Cos-7 cells causes an increase of ER–mitochondria contact sites and alters lipid and calcium exchanges between ER and mitochondria^3^. Such molecular re-establishment underlying cellular mechanism may consequently contribute to the ER and mitochondria morphologic defects as well.

Both the ER and mitochondria provide a membrane source for the formation of the phagophore, the initiation of autophagy^41^. Thus, the defects in those organelles may aggravate the autophagic-dependent pathology. Taken together, these data suggest that diverse cellular dysfunctions caused by *VMP1* deficiency may collectively contribute to the mDAergic neuron loss. In particular, cell death may be processed by necroptosis via the RIPK3 phosphorylation pathway. RIPK1 and RIPK3 are both critical for forming the necrosome, a cytosolic complex required by necroptosis signaling. Dramatically concentrated p-RIPK3 was characterized in our *VMP1*^*cKO*^ mice, indicating that *VMP1*-deficiency might promote the generation of necrosome. However, the linkage between autophagy induction and necroptosis still remains to be determined. Based on previous findings^42^, we speculate that the newly assembled necrosome may be associated with autophagosome membranes. Since a large amount of autophagosome was found to be aberrantly accumulated in *VMP1*^*cKO*^ mice, which may trigger more necrosome forming and promote the necroptosis process. However, further detailed biochemical and IFC investigations on how autophagy interacts with necroptosis are needed in the future. Mechanistically, necroptosis is typically not associated with the activation of caspases^24^. However, in our *VMP1*^cKO^ model higher activity of cleaved-caspase3 (Asp175) was characterized, suggesting apoptosis might cross talk with necroptosis upon the pathological process^43^. Therefore, underneath the cellular context, necroptosis and apoptosis may co-operate in the balanced interplay that involves autophagy.

In neurons, autophagy plays an essential role in the maintenance of axonal homeostasis, manifested by massive autophagosomes predominantly formed in the distal axons^44^. Whereas in our *VMP1*^cKO^ mice the aberrant axonal swollen is so striking, suggesting that the impairment in the autophagic system may cause severe axonal transportation disturbance. Interestingly, a similar phenotype has been reported in the mice with *Atg7* deficiency as well^45^. Moreover, these enlarged puncta extensively harbor α-syn inclusions, consistent with the previous report that in the brain of *GBA*^*−/*−^ medaka α-syn inclusions were accumulated in the axonal swellings^46^. However, in contrast to the terminals α-syn expression, it is barely detectable in the soma of *VMP1*^cKO^ mDAergic neurons. Comparably, in *Atg7* conditional KO mice α-syn was aggregated in the swollen axons of TH^+^ neurons, but not in their cell bodies^47^. These data suggest α-syn may aggregate first in the axon rather than in the soma because of the axonal transportation disturbance^48^. It has been reported that α-syn fibrils are actively transported along microtubules both in the anterograde and retrograde directions^49–51^. We thereby speculate that the reduction of cytoplasmic α-syn in the soma may result from the defects of microtubule-based transport on terminals during the process of pathology.

In summary, *VMP1* deficiency in the mDAergic neurons causes motor disorders, severe mDAergic neuronal loss, mitochondria abnormalities, and autophagy flux disruption. Strikingly, accumulation of α-syn inclusions were extensively identified in the enlarged striatal terminals of *VMP1*^cKO^ mice, suggesting that VMP1 plays an essential role in the microtubule-based transport and ER–Golgi trafficking mediated by autophagy. It is speculated that the *VMP1* deficiency in the nigro-striatal pathway may cause the misfolding protein aggregation in the terminals and eventually trigger the mDAergic neuron degeneration. Taken together, our findings reveal a novel role of VMP1 in modulating neuronal survival and maintaining axonal homeostasis, which suggests that our *VMP1*^cKO^ mice may serve as a useful preclinical model to elucidate the mechanisms of mDAergic neurodegeneration induced by autophagy impairment.

## Supplementary information

Supplementary_legend

Supplementary_Figure 1

Supplementary_Figure 2

Supplementary_Figure3

Supplementary_Video_1

Supplementary_Video_2
