# Supplementary material for: Essential role for autophagy protein VMP1 in maintaining neuronal homeostasis and preventing axonal degeneration
Source: Cell Death Dis. 2021 Jan 22;12(1):116. doi: 10.1038/s41419-021-03412-5 (PMC7822891; doi:10.1038/s41419-021-03412-5)
Supplement: Supplementary file 1 — Supplementary_legend [file 41419_2021_3412_MOESM1_ESM.docx]

**Supplementary Fig. 1 Construction for *VMP1*^fl/fl/ DATCreERT2^ mice and weight record of *VMP1*^cKO^ and *VMP1*^cWT^ mice. A** The basic strategy for TAM-inducible Cre/loxp-directed *VMP1* knockout in the cells where Cre recombinase is active. **B** The recorded body weight of *VMP1*^cKO^ and *VMP1*^cWT^ mice every other day (N=24 mice per genotype). **C** The physical shape of mice as shown. Data were analyzed by using two-way ANOVA followed by Sidak's multiple comparisons test and were represented as mean ± SEM. ****p* < 0.001. mutER, mutant estrogen receptor.

**Supplementary Fig. 2 TH protein expression levels in the brain sections. A** WB analysis displayed TH protein expression levels in the midbrain and striatum (STR). GAPDH as an internal reference. **B** Quantification of TH relative to GAPDH was shown (N=3 mice per genotype). Data were analyzed by using two-way ANOVA followed by Sidak's multiple comparisons test and were represented as mean ± SEM. **p* < 0.05.

**Supplementary Fig. 3** **qRT-PCR analysis of ER-Golgi-transport-related genes.** Data were analyzed by using two-way ANOVA followed by Sidak's multiple comparisons test and were represented as mean ± SEM. **p* < 0.05, ***p* < 0.01.
