# Supplementary figures and images for: Essential role for autophagy protein VMP1 in maintaining neuronal homeostasis and preventing axonal degeneration
Source: Cell Death Dis. 2021 Jan 22;12(1):116. doi: 10.1038/s41419-021-03412-5 (PMC7822891; doi:10.1038/s41419-021-03412-5)

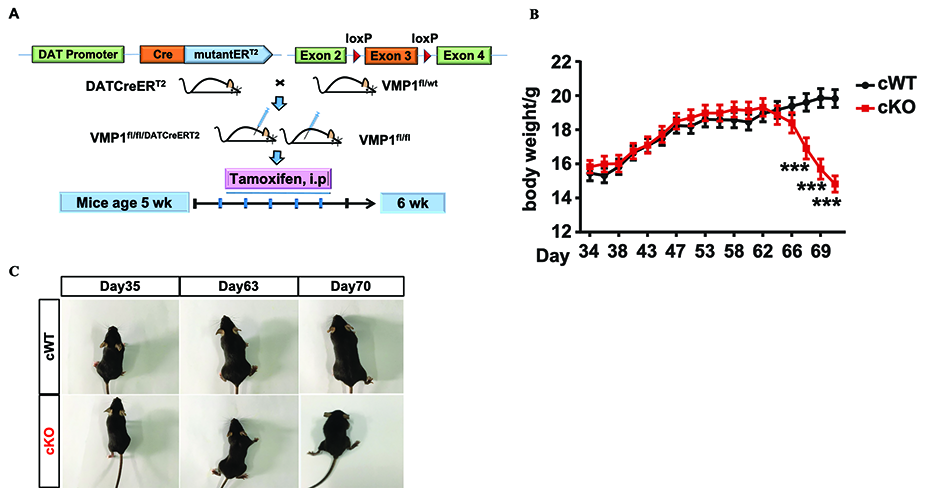

Supplement: Supplementary file 2 — Supplementary_Figure 1 [file 41419_2021_3412_MOESM2_ESM.tif]

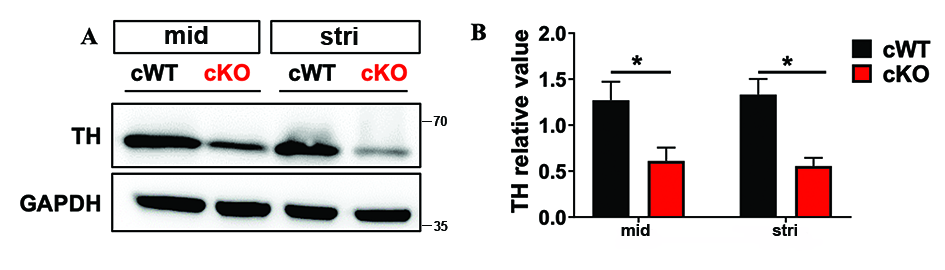

Supplement: Supplementary file 3 — Supplementary_Figure 2 [file 41419_2021_3412_MOESM3_ESM.tif]

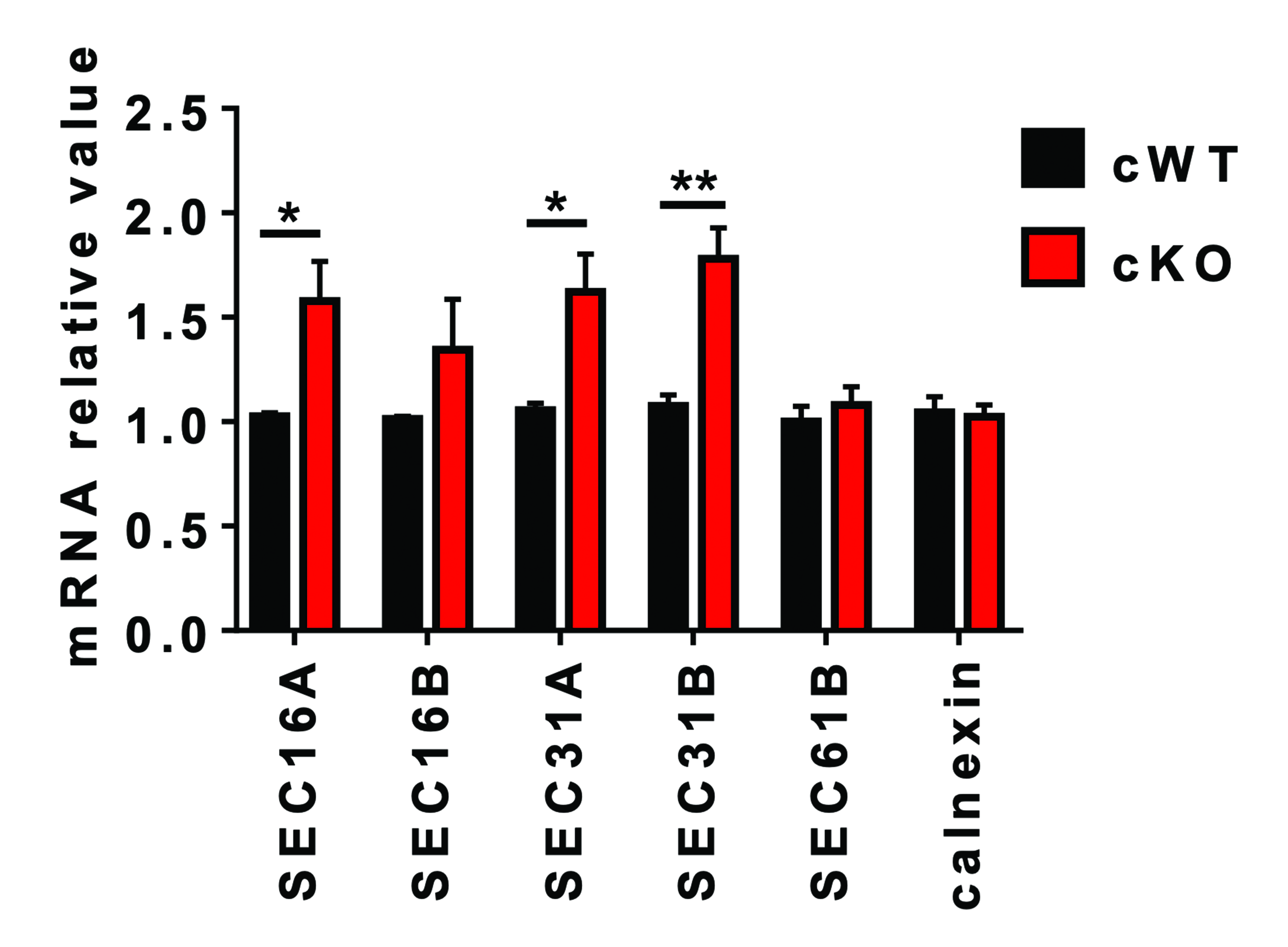

Supplement: Supplementary file 4 — Supplementary_Figure3 [file 41419_2021_3412_MOESM4_ESM.tif]
